# Human Papillomavirus Prevalence in Oral and Oropharyngeal Rinse and Gargle Specimens of Dental Patients and of an HIV-Positive Cohort from Pretoria, South Africa

**DOI:** 10.1155/2020/2395219

**Published:** 2020-08-26

**Authors:** Neil H. Wood, Koketso S. Makua, Ramokone L. Lebelo, Nina Redzic, Ina Benoy, Olivier M. Vanderveken, Johannes Bogers

**Affiliations:** ^1^Department of Periodontology and Oral Medicine, School of Oral Health Sciences, Sefako Makgatho Health Sciences University, Pretoria, South Africa; ^2^Department of Virology, Sefako Makgatho Health Sciences University, Pretoria, South Africa; ^3^HIV and Hepatitis Research Unit, National Health Laboratory Service, Department of Virology, Sefako Makgatho Health Sciences University, Pretoria, South Africa; ^4^Faculty of Medicine and Health Sciences, Applied Molecular Biology Research Group (AMBIOR), Laboratory of Cell Biology and Histology, University of Antwerp, Antwerp, Belgium; ^5^Faculty of Medicine and Health Sciences, University of Antwerp, Antwerp, Belgium; ^6^Department of ENT, Head and Neck Surgery, Antwerp University Hospital, Edegem, Belgium

## Abstract

**Introduction:**

Studies on HPV prevalence in the head and neck region of South Africans are sparse. Of the available reports in the literature, there were no studies on the association between HPV-DNA presence in the mouth and oropharynx in relation to high-risk behaviours such as oral sex practice or tobacco and alcohol use.

**Materials and Methods:**

Following ethical clearance and informed consent, patients attending a regional HIV-management clinic and patients attending a dental hospital were recruited to this study. The participants completed an interview-based questionnaire obtaining demographic information, data on HIV serostatus, and behavioural data including sexual practices and tobacco and alcohol use, and a rinse-and-gargle specimen was taken. Specimens were analysed for HPV DNA on 3 separate PCR/qPCR platforms. Statistical analyses were performed for associations between the study group and categorical variables, HPV status, and data from the questionnaires.

**Results:**

Of 221 participants, 149 were from a general population and 72 from the HIV-management clinic. Smokers comprised 29.4% of the sample, and 45.2% of participants reported to have ever used alcohol. Open mouth kissing during teenage years was confirmed by 64.7% of participants, 40.3% have given oral sex with their mouth, and 44.8% confirmed to have received oral sex from their partner's mouth. Seven participants (3.2%) had detectable *α*-HPV DNA, and 1 (0.4%) had detectable *β*-HPV DNA in their rinse-and-gargle specimens. Two participants were from the HIV-management clinic and 6 from the general dental population (overall 3.6%).

**Conclusion:**

Five high-risk HPV, 2 low-risk HPV, and one *β*-HPV types were detected. The low prevalence of 3.6% compares well to similar studies in different cohorts studied in South Africa and falls within the global oral/oropharyngeal prevalence spectrum. Only 4 participants, all from the HIV-management clinic, had palatine tonsils. No significant relationships were found between HPV presence and demographic data or sexual, oral sexual, tobacco use, or alcohol use, and no associations were seen with numbers of sexual and oral-sex partners.

## 1. Introduction

The human papillomavirus (HPV) is epitheliotropic and requires access to the basal cells of epithelium to initiate a complex sequence of events that additionally relies on specific host reactions and interactions to successfully infect the basal keratinocyte [1]. Mucosal infection by high-risk (HR) HPV subtypes has an established association with an increased risk to develop cervical, anal, penile, and oropharyngeal carcinoma [2, 3].

Taking into consideration the high HIV prevalence in South Africa and the bidirectional risks in acquisition of either infection, HIV or HPV, in cases where the one precedes the other, the importance of providing prevalence data on oral and oropharyngeal HPV infection becomes urgent [2, 4]. The “Human papillomavirus and related diseases” report [5] shows both a deficiency and a comparative reporting lag, in data from Africa when compared to other geographic regions. This is reflected in various systematic reviews conducted on this topic [5–7].

The practice of oral sex (OS), open mouth kissing, having multiple sexual partners, and a compromised immune system increases the risk of acquiring oral/oropharyngeal HPV infection in general population groups [8–12]. The virus spreads through direct contact with infected genital mucous membranes or with bodily fluids. In the South African context, the practice of oral sex, open mouth kissing, smoking, and alcohol consumption has not been adequately studied in relation to HPV infection.

Smoking has been shown to influence HPV clearance from the mouth, and in cases of HPV-positive SCC, prognosis is worsened with concomitant smoking [13, 14].

This study describes the prevalence of HPV-DNA detected in the mouths and oropharynges of a dental clinic population and of an HIV-seropositive clinic population. High-risk sexual behaviours and habits that also include tobacco and alcohol use are reported.

## 2. Materials and Methods

### 2.1. Inclusion of Patients

Patients who attended the University oral health centre and the HIV management clinic were recruited as a convenience sample. The dental patients attended the institution for treatment of oral health-related issues that were unrelated to HPV infection, and the patients attending the HIV clinic came for follow-up and treatment management visits, which were also unrelated to the study.

### 2.2. Data Collection

An interview-based questionnaire was completed prior to oral/oropharyngeal specimen collection. The questionnaire was developed from a previous project [15] and existing literature. Data collected included age, gender, smoking habits, alcohol use, oral sex (OS) contact/practice, and HIV serostatus among others. In the case of the HIV-seropositive cohort, the most recent CD4+ T cell count and HIV viral load were recorded from the patient file. The questionnaires were sequentially numbered to ensure anonymity and delinked from patient names, hospital file-numbers, or other possible identifiers.

### 2.3. Specimen Collection and Transportation

Participants rinsed for 15 seconds and then gargled for 15 seconds with 10 ml of saline and then spit the contents into a Thinprep® vial containing Preservcyt® solution. The specimens were stored at 4°C until they were transported to the laboratory. The material in the vial is fixed by Preservcyt® and can be preserved for 6 months at 15–30°C. For the purposes of this report, oral wash implies sampling of the oral and oropharyngeal mucosae. The vials were numbered with the corresponding questionnaire.

### 2.4. HPV Testing

All oral washes were tested for the presence of HPV with 3 different assays: Abbott HR-HPV assay and the RIATOL qPCR HPV genotyping assay, both focussing on the presence of *⍺*-papilloma viruses, and the AML *β*-human papillomavirus typing assay.

The Abbott *m*2000™ RealTime system was used for the qualitative HPV detection with the HR-HPV assay. This real-time multiplex PCR detection kit reports only limited HPV DNA typing results: presence of HPV 16, HPV 18, and other HR-HPV (pooled signal derived from the following HR-HPV types: 31, 33, 35, 39, 45, 51, 52, 56, 58, 59, 66, and 68). The assay is optimized using probe specificity design and an internal human beta-globin control, which is a sample validity control for cell adequacy, sample extraction, and amplification efficiency. The assay is clinically optimized for cervical cancer screening. This clinically based assay cut-off is also used in this study for the detection of HR-HPV in oral washes.

The Riatol qPCR HPV genotyping assay is an ISO certified, fully automated, clinically validated laboratory-developed test [16]. This qPCR HPV DNA test not only detects 14 HR-HPV types (HPV16 E7, 18 E7, 31 E6, 33 E6, 35 E6, 39 E7, 45 E7, 51 E6, 52 E7, 56 E7, 58 E6, 59 E7, and 68 E7) but also reports selected potential high-risk HPV types (HPV 53 E6, 66 E6, and 67 L1) and two low-risk (LR) HPV types (HPV6 E6 and 11 E6). Cellularity control was performed on every sample, by amplification of the beta-globin gene. Based on the beta-globin standard curve, DNA concentration (ng/*μ*l) was determined in every sample. Samples with a DNA concentration below 0.12 ng/*μ*l are considered as invalid and reported as inconclusive. Type-specific HPV positivity is defined as having a positive amplification signal for that specific HPV type, independently of the beta-globin signal.

Specimens were also tested for a spectrum of *β*-papillomaviruses with the AML lab-developed assay. The assay involves a TaqMan real-time PCR containing type-specific primers and consensus probes capable of detecting multiple *β*-HPV types. In total, 5 multiplex consensus reactions are performed: three with double targets detecting HPV types 1/63, 3/10, and 7/41 and two with triple targets detecting HPV types 2/27/57 and 4/60/65.

### 2.5. Statistics

Sample size estimation was based on the estimation of the proportion of HPV-positive patients in each study group, taking the general global and locally reported prevalence rates into consideration. Based on an overestimated HPV prevalence of 20%, 5% precision, and the 95% confidence level, a sample size of 246 would be required for each group [17].

The Χ^2^ test was used to assess the relationship between the study group and categorical variables. Fisher's exact test was used for 2 × 2 tables or where the requirements for the Χ^2^ test could not be met. The strength of the associations was measured by Cramer's V and by the phi-coefficient, respectively.

The relationship between study groups as well as between age and lifetime sex partners was assessed by the *t*-test (or ANOVA for more than two categories). Data analysis was carried out using SAS (version 9.4 for Windows). The 5% significance level was used.

### 2.6. Ethical Approval

This study received ethical clearance from the Sefako Makgatho Health Sciences University Research and Ethics Committee (SMUREC/P/86/2016). Individuals received an information sheet and provided informed consent prior to participation. Participants had the opportunity to ask questions and not one person was disadvantaged or prejudiced in any way in the event of opting not to participate in the study.

## 3. Results

A total of 221 participants were enrolled, 149 from the general population attending the dental clinic, and 72 attending the regional HIV-management clinic. The actual sample sizes of 72 (HIV clinic) and 149 (dental clinic) used in this study correspond to a precision of 9.2% and 6.5%, respectively (rather than 5.0%), which could be a limitation of the study. For a sample size of 221, this study has the resolution to determine differences between these two datasets. The power value informed the degree of confidence to which our sample size is sufficient in order to see differences between these datasets. A power value of 90% is deemed to be an acceptable level of confidence. Based on these analyses, for a sample size of 221 with a comparison difference of 0.018, the power value is 97.8%. The sample size is, therefore, sufficient to determine any differences between this dataset and global data.

Participants' ages ranged from 20 to 74 years with a mean age of 43.8 years. Majority of the participants were black and more than half were female (Table 1).

16.3% (*n* = 36) of all participants (29.4%) were current smokers with no significant difference between the clinic groups in the number of cigarettes smoked per day. 54.8% of participants confirmed to have consumed alcohol. Of those who confirmed to have consumed alcohol (*n* = 121), 36.4% consumed alcohol in the week prior to the study and 33.1% reported consuming alcohol during the four months before the study (Table 2). There was no significant difference between the HIV-management clinic and dental clinic participants on the amount of alcohol consumed. Thirteen participants used snuff tobacco (5.9%) (data not shown).

Majority (64.7%) of the participants had open mouth kissed during their teenage years (15–19 years) with a significant difference between the two groups where more dental clinic participants practiced open mouth kissing at a very early age (10–14 years) (Table 3). There was also a significant difference in the number of people who practiced open mouth kissing in the past 2 years between the two groups, with data showing that some HIV-positive people did not perform kissing in the past 2 years. Of all the participants, 65.2% (*n* = 144) confirmed they knew what OS was, and 40.3% (*n* = 89) reported to have given OS with their mouths and 44.8% (*n* = 99) reporting to have received OS. Only one of these men tested positive for oral/oropharyngeal HPV and was from the dental clinic group who indicated he did not give nor receive OS from his single OS partner during the past year. There was a significant association between age and number of lifetime OS partners within the dental clinic group (*n* = 145; *p*=0.006) (Table 3). Interestingly, the mean age of those with no OS partners was older than that for those with OS partners.

The HIV-management clinic represents a population of exclusively HIV-positive patients, whereas 4.0% of the dental clinic attendees self-reported to be HIV positive. The HPV (*α*-papillomavirus) prevalence for the dental clinic population (*n* = 149) analysed by the Riatol assay only was 1.4% (95% CI: 0.2–4.9%) (*n* = 2) compared to the 2.8% (95% CI: 0.3–9.8%) (*n* = 2) for the HIV clinic (*n* = 72) with no significant difference between these groups. All specimens except 2 had a positive beta-globin control. Only 3 participants from the dental clinic and none from the HIV clinic had HPV-DNA detectable by the Abbott platform. The combined proportion of oral/oropharyngeal *α*-HPV-positivity for this study (*n* = 221) was 3.2% (*n* = 7) (Table 4). This compares favourably to international reports.

Palatine tonsils were absent in 100% of the dental clinic participants group and in 94.4% of the HIV-clinic participants group. None of the *α*-HPV-positive participants (*n* = 7) had tonsils. Not a single participant presented with HPV-associated lesions during clinical examination.

Table 5 shows the 8 participants that were positive for HPV, 2 from the HIV clinic, and 5 from the dental clinic had detectable *α*-HPV DNA (Table 5). One participant, from the dental clinic, had detectable *β*-HPV DNA. The discrepancy between these two HPV tests is generally seen in HPV assays where the overall concordance is rather low, especially in low-viral load samples (as here). No significant associations were found between HPV presence and any demographic factor or high-risk behaviour or with number of sex or of OS partners (supplementary file).

## 4. Discussion

There are limited data describing the spectrum of HPV genotypes infecting the oral and oropharyngeal mucosae of the general South African population, and there are similarly no reliable data describing the types of HPV present in oral/oropharyngeal HPV-associated benign and malignant lesions of South African populations. Previous studies from South Africa on oral/oropharyngeal HPV infection were further limited by smaller specific cohorts [18–22]. The authenticity of the lower overall prevalence rate (3.2%) found in this study is further supported by other independent studies from South Africa [18, 22, 23].

In general, HPV prevalence studies are centred around the *α*-papillomaviruses because of the strong oncogenic association and transduction potential of E6/E7 oncoproteins. However, it is not strange to find HPV from other genera in the mouth and oropharynx and some even associated with benign lesions and more recently, potentially malignant lesions [24–26]. However, Agalliu and coworkers [27] suggested that HPV other than *α*-papillomaviruses may have a part to play in the aetiology of head and neck SCC, being the first to report a positive association between infection with *β*- and/or *γ*-HPV subtypes. Only one participant from this study had demonstrable *β*-HPV DNA, and we can only speculate that this was a passenger-infection (background infection) as described in the literature [19, 21].

Although this study did not interrogate multiple HPV-types in the individual specimens, it did look at HPV-16, HPV-18, and other HR-HPV types. The finding of multiple types infecting a single individual is not infrequently reported. It is entirely possible for one patient to have two HPV types infecting the same mucosal field and also possible for one patient to have one type affecting more than one mucosal field, e.g., orogenital as evident by numerous concordance studies for different anatomical locations lined by mucosa. No incidental finding of multiple HPV-type infections were made, and the 8 HPV-positive specimens in this study all had single HPV subtypes detected as determined by the three independent assays.

Oral and oropharyngeal HPV prevalence studies report prevalence rates of up to 10.0% for patients considered otherwise healthy. Many of these do not present data on HPV genotypes. In the USA, prevalence of oral HPV infection in a group of 14–69 years old participants was 6.9% and higher among men. The study showed a HR-HPV prevalence of 3.7% and a low-risk HPV prevalence of 3.1% [28]. Another US study demonstrated oral HPV infection prevalence of 2.4% in 18–30 year olds and confirmed the oral-to-oral and oral-to-genital transmission routes of HPV [29]. These fall within the range of a 2010 meta-analysis of oral HPV prevalence globally, which showed a prevalence of under 5.0% across different demographic strata [30]. The combined prevalence of 3.2% found in this study is within this spectrum.

A Swedish study of oral HPV infection in youths between the ages of 15 and 23 years demonstrated higher HPV prevalence rates for males and females at 9.3% and 9.5%, respectively. However, females with concomitant cervical HPV infection had significantly higher oral HPV prevalence rates (17.1%) when compared to those without cervical HPV infection [31]. South African studies on HPV prevalence in the mouth and oropharynx are limited to 14. Of these, only 2 were conducted from oral rinse and gargle samples [22, 23], 6 used oral brushes to collect samples [18, 20, 32–35], and 6 were performed on formalin-fixed paraffin-embedded SCC samples [19, 21, 36–39]. This highlights the importance for data on HPV infection in the head and neck region of South Africans.

An investigation of oral/oropharyngeal HPV presence in 128 South African male factory workers revealed an oral/oropharyngeal HPV prevalence of 5.6% [23]. Chikandiwa and colleagues [22] recently demonstrated an oral/oropharyngeal HPV prevalence of 1.8% in a male population from Cape Town in South Africa. These compare favourably with the prevalence of 3.2% found in this study. Evidence for oral-genital HPV transmission from a South African study reveals oral/oropharyngeal HPV prevalence of 15.0% for the 34 adults examined but unfortunately did not analyse HPV subtypes detected [33]. A novel South African pilot study was in agreement with the Swedish data: when the oral and cervical mucosae were examined for HPV presence in 30 HIV-seropositive women, and Richter and coworkers found that oral mucosal HPV presence was higher (20.0%) in those HIV-positive females with concomitant cervical HPV infection [20]. The authors used a brush-biopsy technique to collect the samples. In contrast, Meyer et al. [40] found no significant difference in the prevalence rates of oral HPV infection when comparing women with cervical HPV infection (5.7%) to those without (5.1%).

The group led by Mbulawa [34] proposed that in the African setting, oral/oropharyngeal HPV is acquired from sexual partners, but in woman, it may also be due to autoinoculation. The authors reported oral HPV infection in 8.4% of study participants. Furthermore to this, it is conceivable that when using saliva as a lubricant, autoinoculation and cross infection with HPV may occur [41].

High-risk sexual practices such as unprotected sexual intercourse, having multiple sexual partners, and the practice of oral sex/anal sex need to be investigated concomitantly with oral/oropharyngeal HPV prevalence because these are classic risk factors for HPV transmission. However, data on oral-sexual practice from South Africa are very limited [17], and no information relating this behaviour to different types of HPV infecting the oral and oropharyngeal mucosae in South Africans exists [23]. The proportion of participants who reported to have ever practiced oral sex were 46.3% for the dental clinic attendees and 50.0% for the patients attending the HIV management clinic (47.5% combined). This is much higher than a previous report published from the same area, which revealed that 22.4% of participants practiced oral sex [15]. In this current study, no link between the practice of oral sex and HPV presence could be demonstrated following analysis of the data in part because of the extraordinary low prevalence of HPV detected.

Global data suggests a much higher oral/oropharyngeal HPV prevalence for HIV-seropositive patients; and HPV infection rates of these mucosal sites are proportional to the level of immune suppression [33]. This study does not align with these global data in that only 2/72 (2.7%) HIV-positive participants had detectable HPV, and none had any clinically evident HPV lesions. This was no different when compared to the general dental participants, in which 6/149 (4%) had detectable HPV (Table 5). Oropharyngeal HPV infection rates are up to 3 times higher in HIV-seropositive patients than in patients with oral SCC [42]. Conversely, it is also reported that the risk for HIV infection is increased in instances of a preceding HPV infection [4]. The natural course and progression of HPV infection also appears to be different to that in HIV-negative individuals due to the HIV-induced immune alteration [43].

It is still unknown whether early or later HPV infection plays any significant role in the mouth/oropharynx nor has latency of HPV in humans been established conclusively [44]. However, several reports of immune reconstitution inflammatory syndrome following highly active antiretroviral treatment have been described with clinical manifestations of HPV infection that include rampant proliferation of oral and perioral papules and wart-like projections [43, 45, 46]. In the HIV-seropositive cohort of this study, no HPV-associated oral lesions were identified, and an oral/oropharyngeal HPV prevalence of 2.8% (2/72) in this particular cohort was found.

Tobacco and alcohol use are considered the traditional risk factors in the development of oral/oropharyngeal squamous cell carcinoma. However, a slow decline in tobacco use globally has also been accompanied by a rise in oropharyngeal cancer incidence [47]. A distinct subset of oropharyngeal HPV-induced carcinomas is already recognized and is increasing in prevalence with HPV-16 seemingly responsible for 40–60% of these SCC's [48–50]. The odds of developing oropharyngeal SCC rises more than 13 times when HPV 16 is found in keratinocytes shed from the oral/oropharyngeal mucosa [49]. Oral SCC incidence in South Africa is reported as 2.7/100000 and SCC of the pharynx as 2.4/100000 [33].

In a report on trends regarding tobacco use in South Africa, the prevalence of smoking among black South Africans was 22.7% and 36.6% among Caucasians; and the trend reflected a decrease in smoking [21, 51]. Another South African study from a different geographic area reported 19.7% of participants to be smokers, and 6.4% used snuff tobacco [15]. This study reports 29.4% of participants who indicated to have ever used tobacco and 16.3% to be current smokers. Thirteen participants (5.9%) used snuff. Aside from delaying HPV clearance, it is also known that smokers with concomitant HR-HPV infection in oropharyngeal SCC have a significantly higher risk of disease recurrence than nonsmoking counterparts [13].

HPV infection has a stronger association with tonsillar SCC than SCC of the mouth [20]. The dispersed reticular network of epithelial cells found in the tonsillar crypts expresses a primitive differentiation. These CD44-positive and nerve growth factor receptor- (NGFR-) positive cells have been shown to have a higher proliferative potential than regular mucosal counterparts. These proliferating cells also express CK5, which is a basal cell marker. The microstructural arrangement of these cryptal epithelial reticulations leads to the cells being exposed to the external environment, making it easier for HPV-access. No microlaceration or wounding is required. The proof of principle for this was published by Kang and colleagues [52]. However, the influence of cryptal surface progenitor cells has not been well-studied with regards to maintenance of cryptal epithelial cells. Similarly, very little information exists on the influence of HPV on the different epithelial cell population found in the tonsil [52].

An interesting incidental finding from this study's population groups showed that 98.2% of participants had their palatine tonsils removed. None of the oral/oropharyngeal HPV prevalence studies we interrogated included data on the presence or absence of palatine tonsils. It is, therefore, conceivable that the absence of tonsils could play a preventive role in HPV-associated SCC of the head and neck. This needs further investigation.

It could be that the oral cavity serves the passenger infection model as a reservoir to inoculate the tonsils and other more distant mucosal sites [19, 21]. The “oral HPV reservoir theory” was also postulated in the context of HIV-seropositive adults by Fatahzadeh and colleagues [53]. In order to further advance the knowledge frontier on the natural course and impact of oral/oropharyngeal HPV infection, the importance of early detection for prevention, persistence studies cannot be overlooked and must be standardized methodologically. Females receiving the HPV vaccine are believed to receive protection for HR-HPV types currently associated with head and neck cancer. This must be performed in parallel to cost-vs-benefit analyses and determination of optimal vaccine administration time in these cohorts [30]. It remains to be seen if this herd immunity will result in coverage of other high-risk populations such as men who have sex with men and HIV-positive cohorts practicing high-risk behaviours.

## 5. Conclusion

The overall prevalence of oral/oropharyngeal HPV DNA in dental clinic attendees and in attendees of an HIV-management clinic is low in this report. This data compares favourably to other South African papers in different cohort studies and also falls within the expected global prevalence range. One participant presented with *β*-papillomavirus DNA present in the rinse and gargle specimen.

Oral/oropharyngeal mucosal infection by HPV is not implied by the detection of any HPV-DNA in these participants. The possibility that these represent passenger infections that serve to inoculate other anatomical sites lined by mucosa must be considered. Single-site coinfection with multiple HPV types may be detected only as a singular HPV positivity using conventional PCR platforms and should be individually identified through sequencing [21]. A significant finding was that a large portion of the population did not have palatine tonsils, which raises the question whether tonsillectomy is prophylactic in the South African population; however, with such limited data on HPV-positive oral and oropharyngeal SCC, this is a question that remains to be answered. Similarly, the value of vaccination in order to prevent a very distinct subset of head and neck cancer must be studied.

## Figures and Tables

**Table 1:** Demographic data for the study population.

Variable	Category	Overall (*n* = 221)		Group				
				Dental clinic (*n* = 149)		HIV clinic (*n* = 72)		*p* value for the between-group test
		*n*	%	*n*	%	*n*	%	
Gender	Male	101	46.5	67	45.0	34	47.2	>0.99
	Female	116	53.5	78	52.3	38	52.8	
	Unknown	4						

Ethnicity	Black	210	96.8	138	95.2	72	100.0	0.41
	Caucasian	4	1.8	4	2.8	0	0.0	
	Indian	2	0.9	2	1.4	0	0.0	
	Mixed heritage	1	0.5	1	0.7	0	0.0	
	Unknown	4						

The age demographic represented by means, standard deviation, and range								
Age	*n*	*n* = 219		*n* = 147		*n* = 72		0.10
	Mean	43.8		42.7		46.0		
	Std deviation	14.0		15.0		11.5		
	Range	20–74		20–74		23–72		

**Table 2:** Tobacco and alcohol use in both participant groups.

Variable	Category	Overall		Group				
				Dental clinic		HIV clinic		*p* value for the between-group test
		*n* = 221 (%)		*n* = 149 (%)		*n* = 72 (%)		
Ever used tobacco	No	156	70.6	104	69.8	52	72.2	0.75
	Yes	65	29.4	45	30.2	20	27.8	

Current smoker	No	185	83.7	121	81.2	64	88.9	0.18
	Yes	36	16.3	28	18.8	8	11.1	

Number of cigarettes per day-grouped (if current smoker; *n* = 36)	1–3	11	31	8	29.6	3	37.5	0.60
	4–8	14	40	10	37.0	4	50.0	
	9 or more	10	29	9	33.3	1	15.5	
	Unknown	1						

Ever used alcohol	No	100	45.2	69	46.3	31	43.1	0.67
	Yes	121	54.8	80	53.7	41	56.9	

Last alcoholic drink-grouped (if use alcohol; *n* = 121)	1–7 days	44	36.4	28	35.0	16	39.0	0.049 (*V* = 0.22)
	1 week–3 months	37	30.6	30	37.5	7	17.1	
	4 months or more	40	33.1	22	27.5	18	43.9	

Number of alcoholic drinks per week-grouped (if use alcohol; *n* = 121)	1	33	29.7	18	25.7	15	36.6	0.46
	2–3	38	34.2	26	37.1	12	29.3	
	4 or more	40	36.0	26	37.1	14	34.1	
	Unknown	10						

**Table 3:** Distribution of kissing and sexual behaviour in both participant groups.

Variable	Category	Overall		Group				
				Dental clinic		HIV clinic		*p* value for the between-group test
		*n* = 221 (%)		*n* = 149 (%)		*n* = 72 (%)		
Age at first open mouth kiss	10–14 years	20	9.7	17	12.6	3	4,2	0.039 (phi = 0.22)
	15–19 years	134	64.7	81	60.0	53	73.6	
	20–24 years	39	18.8	24	17.8	15	20.8	
	25–30 years	6	2.9	6	4.4	0	0.0	
	30 years or older	8	3.9	7	5.2	1	1.4	
	Unknown	14						

Number of people kissed with open mouth in last 2 years	0	21	10.4	0	0.0	21	29.2	<0.0001 (*V* = 0.46)
	1	85	42.3	61	47.3	24	33.3	
	2–3	52	25.9	36	27.9	16	22.2	
	4 or more	43	21.4	32	24.8	14	15.3	
	Unknown	20						

Age of first sexual encounter	10–14 years	19	9.1	12	8.8	7	9.7	0.81
	15–19 years	126	60.3	81	59.1	45	62.5	
	20 years or older	16	30.6	44	32.1	20	27.8	
	Unknown	12						

Number of OS partners (lifetime)	0	112	51.6	76	52.4	36	50.0	0.64
	1–2	61	28.1	38	25.5	23	31.9	
	3 or more	44	20.3	31	20.8	13	18.1	
	Unknown	12						

Number of partners given OS to (lifetime)-grouped (if lifetime OS partners ≥ 1; *n* = 105)	1	41	43.2	31	46.3	10	35.7	0.59
	2–3	34	35.8	22	32.8	12	42.9	
	4 or more	20	21.1	14	20.9	6	21.4	
	Unknown	10						

**Table 4:** HPV results of oral wash specimens for dental clinic and HIV clinic.

Overall for dental clinic and HIV clinic populations			
Test platform^*∗*^	Category	*n* = 221	%
Abbott HR HPV result	Negative	202	91.4
	Positive (HPV 16 and 2x HPV other)	3	1.4
	Unknown (not run)	16	7.2

Riatol qPCR HPV genotyping result	Negative	213	96,3
	Low-risk HPV positive (HPV 53 and 67)	2	0.9
	High-risk HPV positive (HPV 16 and 35)	2	0.9
	DNA insufficient run	2	0.9
	Nonevaluable (technical error)	2	0.9

AML *β*-HPV genotyping result	Negative	216	97.7
	Positive (HPV group 4/40/65)	1	0.5
	DNA insufficient	2	0.9
	Nonevaluable (technical error)	2	0.9

^*∗*^All specimens were tested on the three different platforms.

**Table 5:** Summary of the HPV-positive participants from both populations (*n* = 221).

Age	Gender	Type identified	Test platform	Population	Smoking current	OS given^*∗∗*^	Alcohol use^*∗*^
31	F	HPV-16	Abbott	Dental clinic		Yes	
66	F	HR-HPV	Abbott	Dental clinic		Yes	
35	M	HR-HPV	Abbott	Dental clinic			
28	M	HR-HPV 35	Riatol	Dental clinic	Yes	Yes	Yes
49	M	HR-HPV 16	Riatol	HIV clinic		Yes	
36	M	LR-HPV 53	Riatol	Dental clinic	Yes		Yes
46	M	LR-HPV 67	Riatol	HIV clinic			
66^†^	M	*β*-HPV 4/60/65	Riatol	Dental clinic			Yes

^*∗*^During the 30 days preceding specimen collection. ^*∗∗*^OS given with participant's mouth, and ^†^only participant where *β*-papillomavirus was detected as either HPV-4, HPV-60, or HPV-65.

## Data Availability

The dataset used to support this study are included in the supplementary file, without restrictions, and named “dataset.xlsx.”

## Supplementary Materials

Supplementary Materials (1) Raw dataset for the population groups included in the study. (2) Supplementary statistical analyses of data regarding high-risk behaviors in the study population.
